# ‘It helps me stay on track’: patient experiences using a mobile app for self-management of systemic sclerosis with a focus on fatigue in women

**DOI:** 10.1093/rap/rkag102

**Published:** 2026-08-13

**Authors:** Yen T Chen, Alexandra E Harper, Mary Alore, Elizabeth Haro, John T Morris, Dinesh Khanna, Susan L Murphy

**Affiliations:** Department of Physical Medicine and Rehabilitation, University of Michigan, Ann Arbor, MI, USA; Division of Rheumatology, University of Michigan Scleroderma Program, Ann Arbor, MI, USA; Division of Rheumatology, University of Michigan Scleroderma Program, Ann Arbor, MI, USA; Michigan Institute for Clinical & Health Research, University of Michigan, Ann Arbor, USA; Division of Rheumatology, University of Michigan Scleroderma Program, Ann Arbor, MI, USA; Department of Physical Medicine and Rehabilitation, University of Michigan, Ann Arbor, MI, USA; Division of Rheumatology, University of Michigan Scleroderma Program, Ann Arbor, MI, USA; Virginia C. Crawford Research Institute, Shepherd Center, Atlanta, GA, USA; Division of Rheumatology, University of Michigan Scleroderma Program, Ann Arbor, MI, USA; Division of Rheumatology, Department of Internal Medicine, University of Michigan, Ann Arbor, MI, USA; Department of Physical Medicine and Rehabilitation, University of Michigan, Ann Arbor, MI, USA; Division of Rheumatology, University of Michigan Scleroderma Program, Ann Arbor, MI, USA; Division of Rheumatology, Department of Internal Medicine, University of Michigan, Ann Arbor, MI, USA

**Keywords:** systemic sclerosis, fatigue, symptom management, mobile app, mHealth, usability, behaviour change, resilience, well-being, qualitative study

## Abstract

**Objectives:**

Fatigue is common and burdensome in people with systemic sclerosis (SSc), significantly impacting daily functioning and quality of life. Yet, it remains inadequately addressed in clinical care. This study aimed to explore participants’ experiences using the RENEW (Resilience-building Energy Management to Enhance Well-being) mobile app as a standalone self-paced intervention without peer coaching for fatigue self-management.

**Methods:**

Semi-structured interviews were conducted with participants with SSc following completion of the 12-week RENEW mobile app intervention. Interviews were analysed using RADaR (Rigorous and Accelerated Data Reduction) technique in combination with thematic content analysis.

**Results:**

Fifteen women were included in the analysis (mean age 56 years ± 10), which reflects the higher prevalence of SSc among women. Among participants, 53% had limited SSc, while the remaining had diffuse SSc or overlap, and 40% had been diagnosed with SSc for less than 5 years (short duration). Four themes were identified: (1) the RENEW app and its features were intuitive; (2) the RENEW app’s content enhanced the learning experience; (3) the RENEW app became a partner to support SSc self-management and (4) refinements to the app could enhance the patient experience.

**Conclusion:**

The RENEW app was perceived as a relevant and user-friendly tool for supporting fatigue self-management in SSc. Findings suggest that a standalone app-based approach may offer a scalable option for fatigue self-management in SSc, while also highlighting areas for refinement to improve engagement and long-term use.

**Trial registration:**

clinicaltrials.gov, NCT07402863.

Key messages

- The RENEW app helped participants manage fatigue through practical strategies, structure and daily self-management support.
- Patient stories and videos helped participants feel understood, validated and less alone with systemic sclerosis.
- Qualitative insights from women with systemic sclerosis inform the design of future digital self-management interventions.

## Introduction

SSc or scleroderma is a life-threatening autoimmune disease characterized by fibrosis and vascular abnormalities that affect multiple organ systems [1]. Among the many symptoms experienced by people with SSc, fatigue is one of the most prevalent and burdensome [2–4]. Research has shown that fatigue affects most people with SSc on daily basis, with more than 80% reporting fatigue [5] and nearly half meeting diagnostic criteria for chronic fatigue syndrome [6]. This symptom is associated with substantial limitations in daily functioning, work, social participation and health-related quality of life [7–10]. SSc-related fatigue is challenging to manage because it is multifactorial and often co-occurs with pain, depressive symptoms, physical inactivity and disease-related manifestations such as gastrointestinal dysfunction or breathlessness [11–15]. As a result, fatigue is rarely addressed in routine clinical care [16, 17], with only 5% of people with SSc receiving targeted interventions [18].

Fatigue management interventions based on cognitive behavioural strategies and individualized exercise programs have demonstrated efficacy in other inflammatory rheumatic diseases [19, 20]. Additionally, systematic reviews suggest that theory-based self-management interventions delivered through mobile health (mHealth) platforms can improve fatigue and related symptoms across chronic conditions [21–23]. In SSc, both print- and internet-based self-management programs have been developed [24, 25]. However, a randomized controlled trial (RCT) of an internet-based program found no significant improvements in self-efficacy or other patient-reported outcomes compared with a control group using an educational book [26]. Although participants reported high satisfaction with the content, the lack of structured goal setting, individualized guidance and ongoing support may have limited its efficacy. More recently, a small single-arm virtual intervention called Fatigue and Activity Management Education in Systemic Sclerosis (FAME-iSS) provided preliminary evidence that virtually delivered fatigue management may be feasible and beneficial in SSc [27]. Participants in FAME-iSS reported improvements in fatigue impact, self-efficacy, confidence and understanding of fatigue. However, these findings require confirmation in more rigorous trials.

To address the need for more structured and supportive virtually delivered fatigue interventions for people with SSc, the Resilience-building Energy Management to Enhance Well-being (RENEW) program was co-designed with patient partners [28]. RENEW is a 12-week intervention that combines a digital platform with one-on-one peer health coaching delivered by trained individuals with SSc. In an RCT of 173 participants with SSc, RENEW demonstrated clinically meaningful improvements in fatigue management, pain interference, depressive symptoms and resilience compared with a waitlist control [28]. A secondary mediation analysis identified resilience as a key mechanism underlying improvements in fatigue and related outcomes [29]. Subsequent mixed methods findings further highlighted the value of peer health coaching, with participants describing it as a source of accountability, structured support and shared understanding [30]. At the same time, peer coaching may present challenges for broader dissemination, as it requires resources for recruitment, training and coordination. Also, access may be limited by scheduling constraints and the availability of trained coaches. In addition, some individuals may prefer a more flexible and self-paced approach without coaching [30]. These findings highlight the need to evaluate more scalable approaches to delivering fatigue self-management interventions in SSc.

The logical next step is to examine the mobile app as a standalone intervention. The app may provide continuous access to evidence-based content while supporting self-paced engagement and reducing reliance on resource-intensive coaching. Although the app was delivered without one-on-one peer health coaching, it retained some human supportive elements through videos recorded by RENEW peer health coaches, along with patient audio clips, patient stories and quotes integrated throughout each module to provide encouragement, validation and practical tips and strategies for managing fatigue in daily life. This study used semi-structured qualitative interviews to explore participants’ perceptions and experiences with the RENEW mobile app as a standalone 12-week intervention for fatigue in SSc, with a focus on usability, relevance and opportunities for refinement.

## Methods

### Study design

This qualitative study explored participants’ experiences with the RENEW mobile app as a standalone 12-week self-management intervention for fatigue in SSc. Semi-structured interviews were conducted after program completion to explore app usability, perceived relevance, engagement and recommendations for improvement.

### Participants

**Figure 1:**
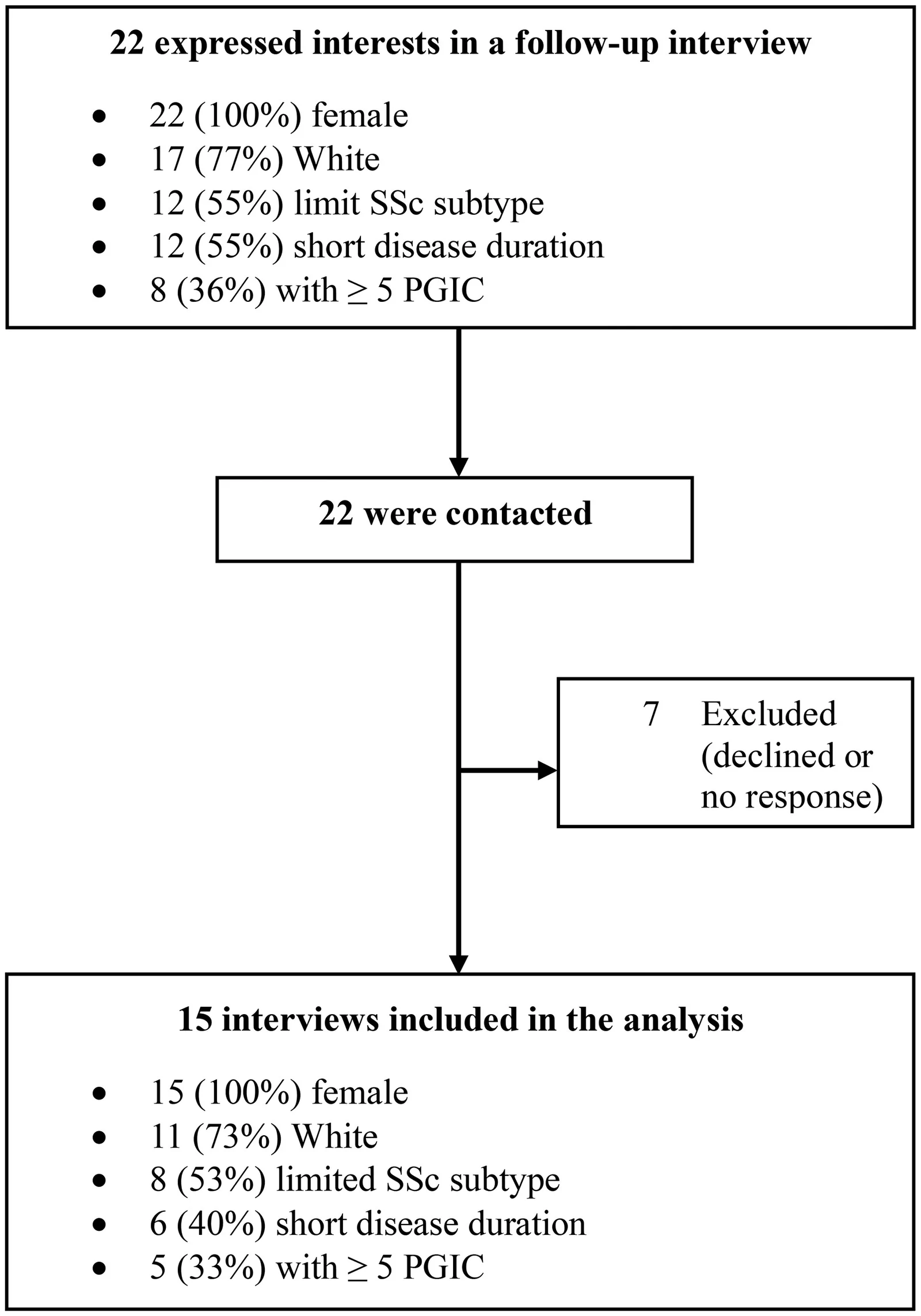
Participant flowchart

A total of 43 participants with SSc enrolled in a 12-week app-alone version of the RENEW program that was delivered without peer health coaching. Participants were recruited between January 2025 and April 2025 through an SSc research registry and SSc social media pages. Individuals who expressed interest were screened by phone for eligibility. Eligible participants were required to be 18 years of age or older, have physician-diagnosed SSc, report moderate to severe fatigue defined as an average score of 4 or higher on the Fatigue Severity Scale [31], have access to a mobile device or tablet and internet connection and be able to speak and read English. Individuals were excluded if they were enrolled in another clinical trial during the study period, had previously participated in the RENEW trial, were receiving rehabilitation or psychological treatment, or had other circumstances that would preclude meaningful participation in study procedures, such as concurrent medical issues or extended travel. At the post-intervention assessment, participants were asked whether they were willing to take part in a brief interview about their experience with the app. Among those who indicated willingness, participants were purposefully invited based on SSc disease duration (≤5 years or >5 years) and Patient Global Impression of Change (PGIC) score [32] to capture a range of intervention experiences. Seven participants who initially expressed interest but later declined or did not respond to the interview invitation were excluded. They were all women (mean age 62 ± 8 years); 6 were White and 1 was Black. Four had limited SSc, 2 had diffuse SSc and 1 had overlap. A subsample was then contacted and invited to participate in interviews conducted between May 2025 and July 2025 (Fig. 1). Interviews continued until no new concepts relevant to the research question were identified in consecutive interviews, resulting in a final sample of 15 participants. The study was approved by the institutional review board at the University of Michigan (HUM00266174), and all participants provided informed consent prior to participation.

### Intervention

**Figure 2:**
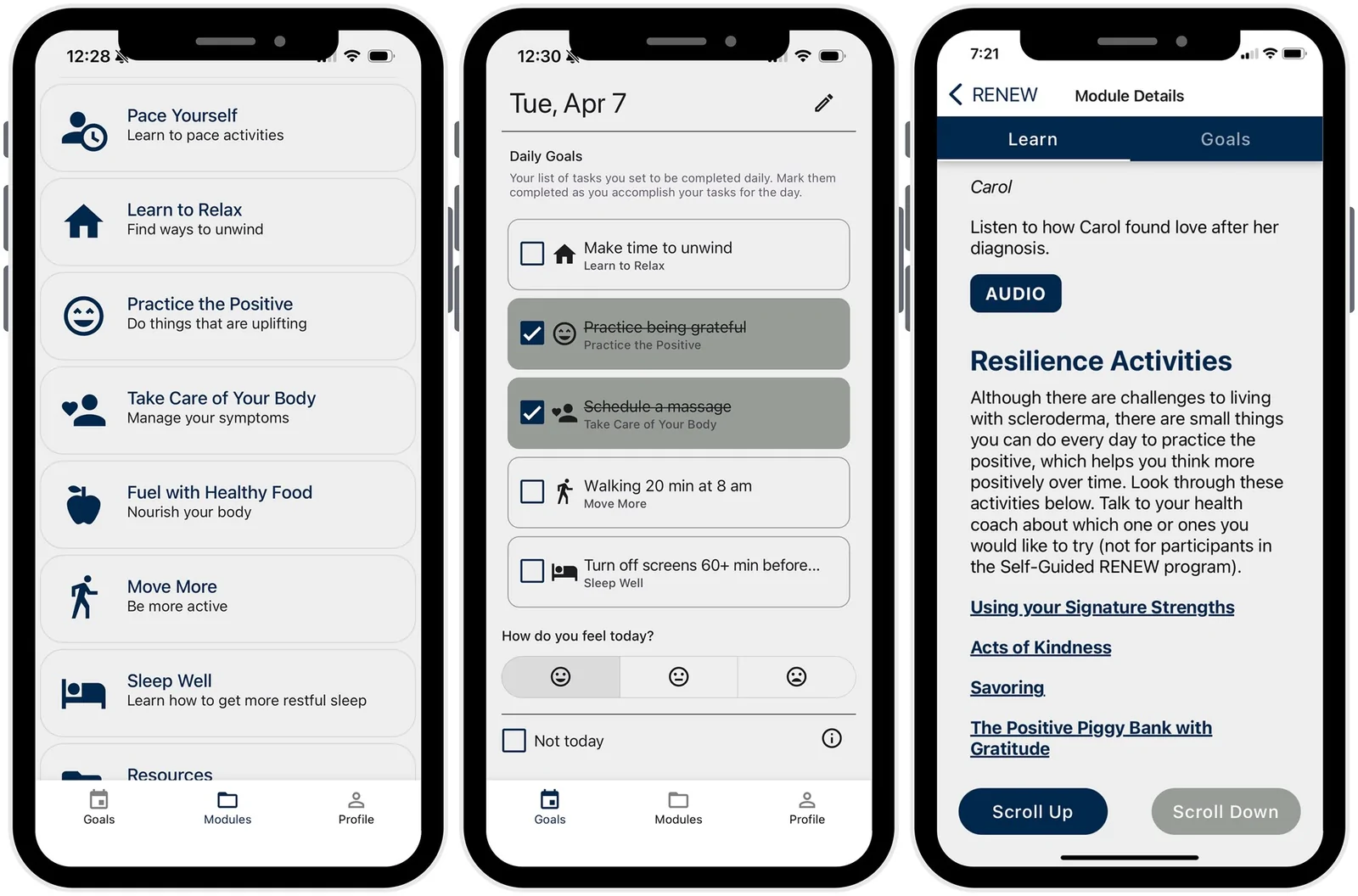
Overview of the RENEW mobile app interface and core features. Key components of the RENEW mobile app include the 7 modules (left screenshot), the goal setting and tracking interface (with daily check-off options), mood rating and a ‘Not today’ option that allows participants to take a planned day off from a goal (middle screenshot), and an example module page (‘Practice the Positive’) features patient audio content and practical exercises that participants can apply in their daily lives (right screenshot)

The intervention consisted of the RENEW mobile app, which delivered evidence-based content adapted from the original RENEW intervention. Grounded in self-efficacy theory [33] and positive psychology [34], the program was designed to support fatigue self-management in people with SSc. The app includes 7 modules focused on activity pacing, self-care, sleep, positive thinking, relaxation, physical activity and healthy diet. Across these modules, participants received fatigue management education, cognitive and behavioural strategies, and practical self-management techniques tailored to daily life with SSc. Participants received detailed instructions for downloading and using the app. During onboarding, they were asked to watch an introductory video recorded by an original RENEW health coach that welcomed them to the program and explained its purpose. The app also included 2 instructional videos, created by original RENEW health coaches, demonstrating how to set healthy goals, particularly for physical activity, and pace daily activities throughout the day to reduce fatigue. Participants were further prompted to view check-in videos at weeks 6 and 12. Ongoing support for questions or technical difficulties was available from the study team throughout the intervention period. Participants were encouraged to use the app independently and at their own pace over 12 weeks. Unlike the original RENEW program, this version did not include one-on-one peer health coaching and was designed to serve as a flexible and self-paced intervention. Figure 2 provides an overview of the RENEW mobile app and its key features.

### Data collection

At baseline, participants self-reported demographic information, including SSc subtype and SSc duration since diagnosis. Fatigue was measured by change in the Functional Assessment of Chronic Illness Therapy-Fatigue short form (FACIT-F) [35], a reliable and validated self-reported measure used in rheumatic disease populations and SSc studies [13]. The FACIT-F includes 13 items that assess fatigue and its impact on daily activities over the past 7 days using a 5-point Likert scale ranging from 1 = not at all to 5 = very much. Raw scores were converted to PROMIS T-scores, with a US population mean of 50 and SD of 10. Higher scores indicate greater perceived fatigue, and negative change scores indicate improvement. Participants completed outcome assessments at baseline, 6 weeks and 12 weeks. At the end of the 12-week intervention, participants were invited to complete a one-on-one semi-structured interview by videoconference (30–45 min). The interview guide was developed by the research team in collaboration with SSc patient partners and included 5 open-ended questions (Table 1). Questions were designed to elicit feedback on key domains, including ease of use, clarity and relevance of content, engagement with app features, perceived benefits and recommendations for future refinement. Interviews were conducted and audio recorded by Y.T.C. and then transcribed verbatim for analysis.

**Table 1:** Interview questions.

1. Overall, how easy or difficult was it to navigate the app and use its features, like setting goals, tracking progress or reviewing modules? • Probing questions ○ What did you think about the design and layout of the app? Were there aspects that could be improved? ○ Were there any specific features that were confusing or challenging to use?
2. Did you experience any technical difficulties while using the app? • Probing questions ○ For example, did you encounter crashes, slow loading or errors? If so, how did these issues impact your experience?
3. How helpful were the modules in addressing your health goals and managing scleroderma-related symptoms? • Probing questions ○ Let’s go over the modules…Were there any topics you felt were missing or needed more detail?
4. Do you see yourself continuing to use the app? Why or why not? • Probing questions ○ What, if anything, would make it difficult for you to continue using the app in the long term? ○ Are there any features or support systems that would make it easier for you to stick with it?
5. What did you like most about the app, and what did you like least? • Probing questions ○ Do you have any additional suggestions for improving the app to better meet the needs of patients with scleroderma?

### Data analysis

Qualitative data were analysed using a codebook approach that combined the RADaR (Rigorous and Accelerated Data Reduction) technique [36] and inductive thematic analysis with no *a priori* theory or framework applied to the data [37, 38]. This approach was selected to support a rigorous, systematic, efficient and iterative analytical process for an interdisciplinary team with varying levels of qualitative research experience. It also allowed the team to balance structured team-based coding with interpretive theme development grounded in participants’ voices [39–41]. Analysis proceeded in several stages. First, Y.T.C., A.E.H., M.A. (a patient partner) and S.L.M. (a senior researcher) familiarized themselves with the data by reading transcripts multiple times. Second, Y.T.C., A.E.H. and M.A. conducted data reduction to remove content not directly relevant to the research question. Coding began after the first 8 interviews had been completed. As additional interviews were conducted, the research team simultaneously analysed the data and met weekly to discuss emerging codes and new findings. Third, Y.T.C., A.E.H. and M.A. independently undertook open coding to identify concepts and ideas related to participants’ experiences with the RENEW app and to develop a preliminary codebook. Once initial codes were generated from the reduced data, Y.T.C. used the preliminary codebook to code the first 25% of the data, and A.E.H. and M.A. independently coded the second 25%. Results were compared and discussed as a team with S.L.M. to reach a consensus on the refined codebook before progressing. Y.T.C. then coded the remaining transcripts, which were reviewed for agreement by A.E.H. and M.A. Thematic saturation was reached after the 12th interview, when no new codes or findings relevant to the research question emerged. To confirm saturation, we completed the remaining 3 interviews that had already been scheduled, resulting in a final sample of 15 participants. The codebook and qualitative findings were reviewed and approved by the multidisciplinary research team, which included a health behaviour researcher (Y.T.C.), occupational therapists (A.E.H. and S.L.M.), a rehabilitation technology research scientist (J.T.M.), a patient partner (M.A.) and a rheumatologist (D.K.). Although some members of the research team were involved in other RENEW studies, the coders had no prior relationship with the participants interviewed in this study as individuals who had previously participated in RENEW were excluded. This multidisciplinary, team-based analytic approach, including the involvement of a patient partner, enhanced the credibility and patient-centeredness of the findings.

## Results

Fifteen participants completed semi-structured interviews. The sample’s mean age was 56 ± 10 years. They were all females, who are much more likely to be diagnosed with SSc than males [42]; 8 with limited SSc subtype and 6 with short SSc duration (≤ 5 years) [43]. Five participants had a PGIC score ≥ 5 (i.e. at least moderately better, and a slight but noticeable change) and 8 participants showed a 3-point improvement in FACIT-fatigue score from baseline to 12 weeks, meeting the minimal clinically important difference for fatigue improvement [44]. Table 2 presents participant characteristics.

**Table 2:** Sample characteristics (*n* = 15).

ID	FSS	FACIT-F baseline	FACIT-F week 6	FACIT-F week 12	FACIT-F change score^a^	PGIC score	Age	Race	SSc subtype	SSc duration
11	5.9	66.9	45.7	45.2	−21.7	7	49	White	Diffuse	Short
108	5.1	69.4	49.3	51.5	−17.9	5	63	Hispanic	Overlap	Long
21	5.8	65.5	64.6	56.5	−9	4	46	White	Limited	Long
49	4.9	67.9	61.3	59.8	−8.1	5	61	White	Limited	Long
19	6.2	59.8	54.0	52.1	−7.7	5	53	Black	Diffuse	Short
123	4.0	51.4	44.9	44	−7.4	1	55	White	Limited	Long
73	5.4	60.8	59.7	55.2	−5.6	3	48	White	Diffuse	Long
83	5.0	63	59.4	57.9	−5.1	3	39	Black	Limited	Short
7	5.9	69.7	68.6	67.2	−2.5	4	60	White	Limited	Short
4	5.8	61.3	55.0	58.9	−2.4	2	68	White	Diffuse	Short
63	5.7	59.9	62.4	57.5	−2.4	5	68	White	Limited	Long
17	7.0	74.1	64.1	72.4	−1.7	4	61	White	Limited	Long
81	6.2	63.6	62.3	63.6	0	4	68	Hispanic	Limited	Long
36	4.0	61.4	60.4	62	0.6	2	54	White	Diffuse	Long
90	6.6	72.1	67.7	74.4	2.3	3	41	White	Diffuse	Short

a To assess change in fatigue, we calculated the difference between the FACIT-fatigue score at baseline and the score at 12 weeks. Negative values indicated a favourable change, suggesting an improvement or decrease in fatigue. FSS, Fatigue Severity Scale. PGIC, Patient Global Impression of Change: 1 = No change (or condition has gotten worse); 2 = Almost the same, hardly any change at all; 3 = A little better, but no noticeable change; 4 = Somewhat better, but the change has not made any real difference; 5 = Moderately better, and a slight but noticeable change; 6 = Better and a definite improvement that has made a real and worthwhile difference; 7 = A great deal better and a considerable improvement that has made all the difference.

**Figure 3:**
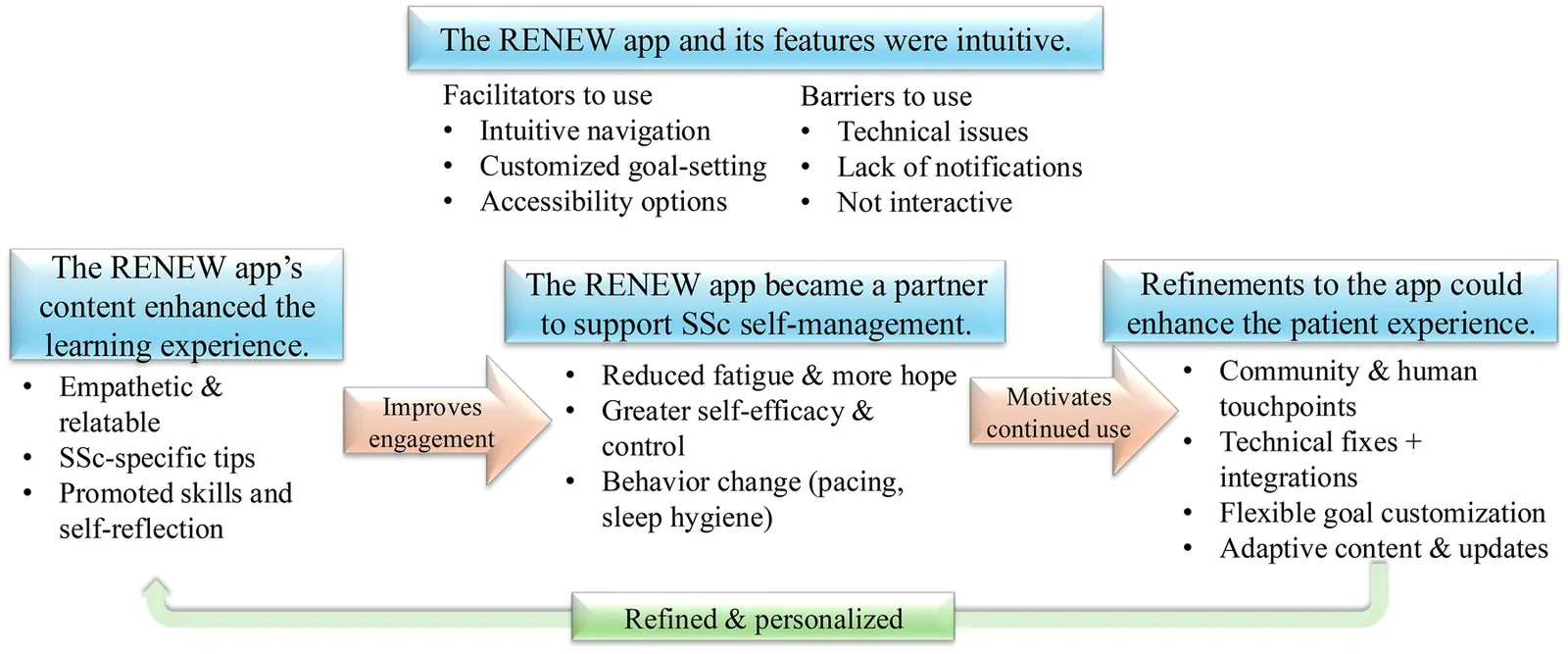
Conceptual model of participant experiences of the RENEW app. Participants reported that the RENEW app’s intuitive features (e.g. customized goal setting and tracking) served as facilitators to use. The content enhanced the learning experience through empathetic and relatable messaging, SSc-specific tips, and promotion of self-reflection skills. Perceived benefits included reduced fatigue, increased hope, greater self-efficacy and perceived control, and behaviour change (e.g. pacing, sleep hygiene). The model also highlights future refinements, such as community touchpoints, technical fixes, flexible goal customization and adaptive content that could enhance sustainability and growth. The app was perceived as particularly beneficial for newly diagnosed patients or who reported limited experience or structure in their self-management

Four main themes emerged: (1) the RENEW app and its features were intuitive, (2) the RENEW app’s content enhanced the learning experience, (3) the RENEW app became a partner to support SSc self-management and (4) refinements to the app could enhance the patient experience. A conceptual model visualizing these themes is depicted in Fig. 3. Themes are presented below with exemplar quotes in Table 3.

**Table 3:** Representative quotes for each theme and subtheme.

Theme 1: RENEW app and its features were intuitive	
Category	Example quote
**Goal setting and tracking were useful**	*There’s a lot of good resources and information all in one place and it’s an easy place just to, like I said, keep track of what you’re trying to do or what you need to do or you want to do a good way to look back and reflect and see what you’ve accomplished and not.* (ID#11)
	*I liked that I could choose my goals I wanted to focus on rather than having goals set for me.* (ID # 21)
	*You check it off (goals) and yeah, it feels like you’ve accomplished something.* (ID #11)
	*I liked that I could set and personalize my own goals and keep track of how I was doing.* (ID #123)
**The app design and interface were user-friendly**	*I thought it was fairly easy and self-explanatory.* (ID # 21)
	*I think the audios and videos made by patients with scleroderma are encouraging and helpful.* (ID #73)
	*There’s a lot of different (formats), if I don’t want to watch a video, I can read about it. If I don’t like to read about it, I can watch the video.* (ID #123)
**Theme 2: RENEW app’s content enhanced the learning experience**	
**Peer health coaches and patient stories were relatable**	*I did like the videos. Those videos they walked you through. The ones that walk you through it so you’re not trying to learn it on your own.* (ID #7)
	*I liked the real stories and the pictures. Honestly, this may sound so weird, but I like to see what people look like with scleroderma because I feel like there’s such a wide range. I just like to see what real people look like dealing with the disease.* (ID #90)
	*What I liked about it probably the most was hearing from other scleroderma patients. I don’t know anyone else who has it. So this time of actually hearing someone I could relate to.* (ID #11)
**Modules contained practical, SSc-specific information missing from other resources**	*I’ve learned how to do some pacing and that’s significantly helped with my fatigue.* (ID #11)
	*Have learned to turn the lights off and just lay there. It takes me a while…I am getting a little bit more sleep than I had been.* (ID #17)
	*It (app) has some really wonderful tips and encouraging people to understand why it’s important, even though you’re in pain to be moving.* (ID #81)
	*I think pace yourself is always a huge one because people are used to being very fast paced. I think for the most part, going about life and just doing whatever, but your energy level changes when you have scleroderma and you really have to manage your time well. And so I think that I really liked the idea of prioritizing because you no longer are going to be able to do everything. I used to be able to go through a house and clean it in a day. I can’t do that anymore. So I actually have a schedule that I’ve built for myself, and I do different things every day. I really liked the prioritizing and then the pace, but primarily a plan to begin with. So I really liked the way this was put together. (ID #81)*
	*It seems as if their scleroderma isn’t as progressive as in mine. And you don’t hear too many people talk about the ulcers. They just kind of talk about how their life has changed, but it is not really directed more to the actual disease.* (ID #83)
	*I have facial tightness very much. So something else that’s a real big issue is dental work because the mouth is so tight it’s very hard to stretch it. So basically, probably get more information with that.* (ID #83)
	*I think the modules were very helpful and I think it also helps having someone, a patient living with scleroderma be the one to speak in those modules. That was important to me.* (ID #73)
**Theme 3: RENEW app became a partner to support SSc self-management**	
**RENEW facilitated physical and psychological benefits**	*I liked hearing other people’s stories. I need the reminders, forget and go into denial a little or like… Accept it, stop challenging it. That’s helpful I think.* (ID #90)
	*This (app) is just helpful for keeping me consistent, keeping me doing something versuss nothing, and holding me accountable to myself, which is what I like.* (ID #108)
	*Before I got sick, I was active, very, very active, and it was hard for me to slow down once I got home. So I ran into all kinds of problems with that. But this helps me to recognize when I need to slow down… That’s what I really liked about this. It helped me to recognize a lot of things about myself that I can improve upon and learn to recognize when I need to start, stop or reduce certain things.* (ID #108)
	*Sleep (module) so, I have loved this module because it’s one of the biggest things that I have…Sleep is my biggest challenge because I think because I’m getting older, my sleep looks different…So I love this module because it allows you to think, okay, how am I going to prepare for my rest? What can I do about it? Behavior tips, timing, bedroom tips, body temperature, environment temperature. So this is your recovery time.* (ID #81)
**RENEW supported behaviour change**	*Manage your energy. That for me was very helpful because I tend to find out that once I have little strength, I try to do everything at once and then I just crash. So pacing myself, I needed to know that pacing myself wasn’t bad. And if I felt tired, I shouldn’t use all my energy that I stored up on just one day or one event. So I found that very helpful.* (ID #19)
	*What I started doing is I started stretching. I stopped using my phone, I stopped using the TV, I would read, or I would just do relaxation stretches, yoga. So there were just different things that I introduced in order to help myself. And it’s really made a difference. And to set a goal is really cool.* (ID #81)
	*The most I would say is how it kept me consistent in what I was doing and it got me into a daily habit of doing things consistently. And it made me come up with goals.* (ID #17)
	*And learning to not say, no…That’s a hill climb. But yeah, I really enjoy this tool.* (ID #108)
**Perceived impact varied by disease duration**	*I found this a very useful app for someone who is just been told they have scleroderma such a great resource for me.* (ID #81)
	*I’ve had the disease for 20 years, so I think that’s a difference. So it’s hard for me to say did I learn a lot with absolutely no disrespect to anybody. But I have researched and lived through as many things. So for me not so much, but I think a new person coming in, it would be very helpful.* (ID #49)
**Theme 4: Refinements to the app could enhance the patient experience**	
**Enhance accessibility (technical aspects) to reduce barriers to use the app**	*For people that do have dry eyes, it’s definitely a recommendation to have more videos or audio it’s a lot of strain on the eyes.* (JL)
	*Makes it where it’s microphone ability, not just typing. Because with Raynaud’s sometimes our phones don’t hit the buttons with our cold fingers.* (ID #7)
**Personalize and expand features to improve perceived value**	*The lack of flexibility with being able to modify goals and make them more interactive…So that was problematic for me. I felt like the goal check boxes were too static to be effective* (ID #63)
	*I know I do track my symptoms and water, but I track it in a separate app. But again, if I’m tracking other stuff along with it or even tracking if I took a nap that day, how long was my nap so that I could see is that affecting my sleep at night, looking back at the week and it giving me a little synopsis… Just so I can track week to week if I’m not feeling well, maybe I napped more that week or maybe I drank less water. Some people want to track that. It might be helpful.* (ID #21)
	*All these things are hooked together in my phone…tracking my steps all day long if I wear my Fitbit and if I’m not wearing my Fitbit it’s tracking my walking from my phone, which isn’t quite as accurate but it’s still something.* (ID #63)
	*It would be awesome if the app could pick up the Peloton information and say, oh, KR went in here and she did her, if I did a meditation through their app, she came in and did that today. Or they keep track of your h and what you’ve been doing. I know that’s difficult and there’s copyright and all that stuff to do, but if you shoot for the stars, that would be a really cool thing to integrate.* (ID #49)
	*I couldn’t log in directly and maybe that’s because it was the study, so I had to go through the email and I just found that extremely annoying.* (ID #36)
	*I felt just me personally that there was just a lack of flexibility in tracking activity and sleep and all and fatigue and all that.* (ID #63)
	*If this app had some specific related, just almost like a section of notes with your doctor, like medication, do I need to request medication? Just these little things because the exhaustion, I think a huge part of it is tracking symptoms to be able to hold them long enough to be accurate to give to the doctor for the next appointment. And also just the medication management and tracking which medicines are working. So if it was even just a add your medications here, but to have all that in the app and pull up when I’m at the doctor’s to where I can pull the renew up and say, hold on, I remember more. And then have the note of new symptoms or just kind of a quick cheat sheet that.* (ID #90)
	*With the mental health…on things that people are really bothered by and you just might be able to see some hint, something that might want to be added into the app. (*ID #49*)*
	*The things that you’re supposed to do, the introducing new foods, the what was it like buying stuff that made it easier? I had a hard time with that because I didn’t know what I could buy that would make life easier.* (ID #17)
	*Maybe do the videos for different stretching. Gentle aerobics. You can show videos of what those look like as well as gentle, maybe even seated stretches.* (ID #7)
	*The COVID-19 and scleroderma… if there’s one that approves the list for patients just to have it like, oh, these are the vaccines you need annually. These are recommended by the medical board or whatever.* (ID #90)
	*I think what would’ve been helpful for me is, now you’re diagnosed, what can you expect? What can some patients experience?* (ID #36)
	*What might happen in 5 years, 10 years, or 15 years down the line…include a list of symptoms or changes that people might experience as their condition progresses. I think that kind of long-term perspective could be really helpful for people, especially in planning and knowing what to look out for.* (ID #36)
**Add opportunities for peer connections**	*I think something like ask a question, and then have people be able to interact with each other and answer even if it’s just one thing, an interactive question as part of the app.* (ID #4)
	*I do think that perhaps having a peer on call or I don’t know, somehow having somebody to talk to, somebody to talk to one-on-one or maybe in a small group early on would be helpful as an augmentation of the information on the website and doing that app.* (ID #63)
	*I would really love a community. Just one of those things where it starts to build itself and there’s maybe a monthly zoom conference just for everyone to, I don’t know, stay five min of whatever if they want to or not if they don’t, but just to have a platform… Even people who are around people all the time during the day, it’s still like I’m not going to talk about this kind of stuff, nor would anyone understand what I was saying if I tried.* (ID #90)

### Theme 1: The RENEW app and its features were intuitive

Participants valued app features that made self-management accessible, motivating and flexible. They appreciated that the mobile format placed resources ‘all in one place’, which reduced cognitive load because they did not have to remember where to find information. Participants also valued the goal setting and tracking features because they could choose and personalize goals that were meaningful to them, monitor their progress and feel a sense of accomplishment when completing activities. As one participant shared, ‘I liked that I could choose my goals I wanted to focus on rather than having goals set for me’. Another explained, ‘being able to check things off each day made me feel like I was actually making progress’. Participants also appreciated the app’s multiple content formats, including text, audio and short videos, which allowed them to engage with the material in ways that matched their learning preferences and energy levels. One participant noted, ‘if I don’t want to watch a video, I can read about it. If I am tired, I can just listen to the audios’.

### Theme 2: The RENEW app’s content enhanced the learning experience

Participants described the RENEW app content as relevant, relatable, practical and supportive of learning. They consistently highlighted the importance of peer perspectives. Peer health coach videos that introduced the program and provided examples of how to set healthy goals and use pacing strategies, along with midpoint check-in and wrap-up videos, were especially valued and described as ‘encouraging and helpful’. Patient stories also helped participants feel understood, validated and less alone. As one participant shared, ‘it felt like they really understood what I was going through’. Content that addressed common but less openly discussed challenges, such as unpredictable symptom flares and emotional distress, was especially meaningful. One participant noted, ‘it made me feel seen and not alone in this’. Participants also valued the practical, SSc-specific information included in the modules, which they felt was often missing from other resources. Modules on Pace Yourself, Sleep Well and Take Care of Your Body were described as actionable and directly applicable to daily life with SSc. Participants appreciated strategies that could easily use, such as pacing activities and improving sleep routines. One participant noted that these were ‘things I could actually use in my day-to-day life’.

### Theme 3: The RENEW app became a partner to support SSc self-management

Participants described the RENEW app as an active partner in supporting self-management. They reported increased awareness of their symptoms, activity patterns and how they used their energy throughout the day. Several described feeling less tired, sleeping better and feeling more confident in managing symptoms. In addition, participants explained meaningful shifts in how they interpreted symptom fluctuations, particularly during flares. For example, one participant explained that the program helped them realize that ‘taking it easy during a flare is not a failure, it is what my body needs’. Along with improvements in physical and psychological symptoms, participants noted that RENEW supported behaviour change. The app provided structure and accountability, with features such as daily check-in notifications helping participants follow through on their goals. It also encouraged greater awareness of energy use. As one participant shared, ‘it made me think more about my energy patterns and how I use them’. Other participants reported improved activity pacing, regular stretching, increased hydration and more proactive planning around energy levels. Many described fewer ‘boom and bust’ patterns and a shift towards more sustainable routines. As one participant stated, ‘I am better at pacing now instead of pushing too hard and crashing later’. Although perceptions of RENEW were positive, perceived impact varied by participants’ prior experience managing SSc. Participants who were newly diagnosed or seeking more structure in their self-management routines described the app as highly beneficial and, in some cases, ‘life changing’. In contrast, participants who had already developed well-established coping strategies often viewed the content as reinforcing existing knowledge rather than introducing new approaches. As one participant explained, ‘it reinforced what I already knew, but did not change things dramatically’.

### Theme 4: Refinements to the app could enhance the patient experience

Participants identified several opportunities to enhance the app’s accessibility, personalization and long-term engagement. Accessibility improvements were especially important for those with SSc-related symptoms like dry eyes and limited hand dexterity. Suggested enhancements included less text, more engaging audio or video content, and voice command functionality. As one participant noted, ‘on days when my hands are really bad, it would help to have something like voice control’. Participants also recommended greater personalization, including easier goal editing, symptom tracking, wearable device integration, progress summaries, customizable notifications, a more streamlined login process and a notes section to support medical appointments. To keep the app relevant over time, participants suggested regular content updates with new research and patient stories, beginner and advanced content pathways (to match evolving needs and levels of self-management experience), expanded mental health and disease progression information, SSc-specific nutrition guidance, adaptive exercises (e.g. seated or low-impact exercises) and strategies for managing severe symptoms such as digital ulcers, limited mouth opening and skin sensitivity. As one participant explained, ‘it would be helpful to have more tips for when symptoms are more severe and limiting’. Participants also wanted opportunities for peer connection and human support, such as discussion forums, moderated support groups, brief check-in messages or live Q&A sessions. These additions were viewed as ways to enhance motivation, accountability and social connection.

## Discussion

This qualitative study explored participants’ experiences using the RENEW mobile app as a standalone 12-week intervention for fatigue self-management in SSc, without one-on-one peer health coaching. Participants generally viewed the app as acceptable, relevant and supportive of self-management, while also identifying several opportunities to improve accessibility, personalization and long-term engagement. Importantly, participants described fatigue as closely intertwined with other symptoms. This perspective suggests that interventions targeting fatigue may be most effective when integrated within comprehensive disease self-management programs rather than focusing on fatigue alone. Overall, these findings suggest that a standalone app-based version of the RENEW self-management program may represent a feasible approach to fatigue self-management support in SSc.

Participants generally found the RENEW app intuitive and easy to use, and they valued having fatigue management resources consolidated within a single mobile platform. This centralized format appeared to reduce cognitive burden by minimizing the need to search across multiple sources. This finding is consistent with broader mHealth literature showing that usability, ease of navigation and streamlined design are important for engagement with digital self-management tools in chronic conditions [45, 46]. However, our findings also highlight the need to consider the specific accessibility needs of people with SSc. Although participants responded positively to the combination of text, audio and video content, they also suggested additional features such as voice-command functionality, higher contrast display options and more visually engaging elements. These recommendations likely reflect the physical challenges associated with SSc, including Raynaud’s phenomenon, digital ulcers and limited hand dexterity, which may make standard touchscreen interactions more difficult. In this way, our findings support current rheumatology guidance emphasizing that digital tools should be designed with the needs and limitations of the target population in mind [47, 48]. Notably, the RENEW app was already co-designed with patient partners who provided feedback on button-based navigation to accommodate hand limitations [28]; the current findings suggest that ongoing refinement may be needed to further improve accessibility for users with more severe physical impairment.

Participants consistently described the SSc-specific content as an important part of their experience with the app. Health coach videos and patient stories appeared to enhance participants’ sense of validation and reduce feelings of isolation, particularly when the content reflected challenges that are often difficult to explain to others without SSc. In the context of a rare disease, for which patients may encounter limited understanding of the day-to-day impact of SSc, the empathetic and non-judgmental tone of the app’s content may have provided a meaningful source of psychosocial support. This interpretation is consistent with prior qualitative work in SSc fatigue management, including FAME-iSS, in which participants emphasized ‘the power in our group because we had a common condition’ [27]. It is also consistent with earlier work on RENEW showing that supportive human elements were important to participant engagement [30]. The current findings suggest that disease-specific content delivered through a digital platform, even without a live facilitator, may still foster a sense of connection and validation.

An important finding of this study was that participants described the standalone RENEW app as functioning like a ‘partner’ in supporting self-management, even in the absence of a human coach. Participants reported increased awareness of their energy patterns, greater consistency with activity pacing and a reduction in ‘boom and bust’ cycles of overexertion followed by prolonged fatigue. They also described adopting more proactive strategies, and reframing periods of low activity during flares as ‘a necessary response, not a failure’. These changes were accompanied by perceived improvements in fatigue, sleep quality, hopefulness and confidence in managing symptoms, suggesting that the app may support both behavioural and psychological aspects of fatigue self-management. These findings are noteworthy in light of the original RENEW trial’s acceptability data, in which only a small proportion of participants preferred digital tools alone, while most favoured coaching or a combination of coaching and digital support [49]. In contrast, the current study suggests that when delivered as a standalone intervention, the app may still support meaningful engagement in self-management. One possible explanation is that features such as structured goal setting and automated encouragement reflect key components of self-efficacy theory [33], which were emphasized and reinforced by peer coaches in the previous RENEW intervention that included coaching. This may help explain why participants in the current study were able to engage in behaviour change despite the absence of a live peer health coach. These findings also differ from an earlier internet-based self-management program for SSc that lacked interactive behavioural components and did not demonstrate benefits beyond an educational control [26], suggesting that the more interactive and patient-informed design of the RENEW app may be an important factor in its perceived usefulness.

The perceived impact of the intervention varied according to participants’ prior experience managing SSc. Participants who were newly diagnosed or who reported limited experience or structure in their self-management routines described the app as particularly helpful, especially modules addressing activity pacing, body care and sleep, which they viewed as directly applicable to daily life. In contrast, those with more established coping strategies often found the content reassuring and validating but less likely to prompt substantial behavioural change. Although these differences were often observed among participants with longer disease duration, the variation appeared more closely related to experience with self-management rather than disease duration alone. This pattern is consistent with findings from the original RENEW randomized controlled trial, in which individuals with shorter disease duration experienced greater improvements in fatigue than those living with SSc for a longer period [28]. However, this feasibility study was not designed or powered to compare baseline fatigue by disease duration. It also aligns with prior mixed methods research showing that participants with longer disease duration often desired more tailored and advanced content, including SSc-specific symptom management strategies [30]. These findings suggest that future iterations of the RENEW app may benefit from a more adaptive approach, such as tiered content pathways that provide foundational material for individuals with less self-management experience alongside more advanced modules addressing disease progression, complex symptom management, SSc-specific nutrition guidance, adaptive seated exercises and mental health.

Participants’ desire for peer-to-peer connection and occasional human support highlights an important consideration in digital health interventions. While standalone apps offer scalability and continuous accessibility, the human element remains important for fostering connection and sustaining motivation [50]. In the original RENEW trial, peer health coaching was identified as a highly valued component, with participants emphasizing the importance of shared lived experience and accountability [30]. The current findings suggest that although the app can support some of these functions through structured goal setting and patient-informed content, participants still expressed a desire for opportunities for interaction. Incorporating features such as moderated discussion forums or periodic live question-and-answer sessions may help address this need while maintaining scalability. In addition, future work could explore the use of artificial intelligence to provide personalized, on-demand support within the app. By demonstrating the feasibility and perceived value of a standalone digital platform, this study provides a foundation for evaluating these more interactive and adaptive approaches.

Participants also identified several technical issues that, although not undermining overall satisfaction, created barriers to use. Common challenges included login difficulties, inconsistent reminder delivery and time-zone-related errors. These findings are consistent with broader mHealth literature highlighting the importance of technical reliability for sustained engagement [45]. Participants also recommended integrating wearable devices to support objective activity measurement and adding the ability to export progress summaries. These features may enhance both self-monitoring and communication with healthcare providers. Addressing these technical challenges will be important to ensure that the app functions as a reliable and sustainable self-management tool rather than a short-term educational resource.

### Limitations

This study has several limitations that should be considered when interpreting the findings. First, the current small sample was drawn from a single-arm feasibility trial, and those who volunteered for qualitative interviews may represent a subset of users with higher baseline digital literacy, engagement or satisfaction. Second, this feasibility study did not collect information on medication use or the severity of appearance-related changes. Future efficacy studies could collect both information to better account for their potential impact on intervention outcomes. Third, the interview guide used broad and open-ended questions to explore participants’ overall experiences with the app. Although this approach allowed participants to identify the aspects of the intervention they found most meaningful, it may have limited the depth of information obtained regarding fatigue-specific self-management experiences. Future studies could incorporate additional targeted probes to better understand how digital interventions support fatigue management. Fourth, although formal member checking was not conducted, a patient partner (M.A.) participated in data analysis, interpretation and creation of the conceptual model which helped to enhance the validity and patient-centeredness of the findings. Fifth, the 12-week duration of app use precludes conclusions about long-term engagement and the durability of the reported behavioural changes, an important consideration given participants’ emphasis on the need for sustained relevance. Finally, the predominantly female and White composition of the sample limits the generalizability of these findings to more diverse populations with SSc.

## Conclusion

The RENEW mobile app was generally viewed as a relevant and user-friendly tool for fatigue self-management in people with SSc. Participants described the app as offering disease-specific content, practical strategies and structured support that promoted reflection, self-management and use of coping strategies in daily life. These findings suggest that the RENEW app may have potential as a scalable, standalone approach to fatigue self-management in SSc, while also identifying clear priorities for future refinement. Continued development should focus on accessibility, greater personalization and scalable opportunities for connection and support so that the app can remain relevant to users with differing needs over time.

## Data Availability

The data underlying this article will be shared on reasonable request to the corresponding author.
